# Longitudinal Serum Uric Acid Variation and Risk of Major Adverse Cardiovascular Events and Mortality: A Systematic Review and Meta‐Analysis

**DOI:** 10.1002/clc.70453

**Published:** 2026-09-02

**Authors:** Saima Nisar, Asif Shah, Abida Perveen

**Affiliations:** ^1^ Croydon University Hospital London UK; ^2^ Zakir Khan Shaheed Hospital Matta Swat Pakistan; ^3^ Ibn E Seena Hospital Kabul Afghanistan

**Keywords:** cardiovascular disease, longitudinal variability, major adverse cardiovascular events, meta‐analysis, serum uric acid

## Abstract

**Background:**

Longitudinal variability in serum uric acid (SUA) has emerged as a potential predictor of adverse cardiovascular outcomes beyond a single baseline measurement. However, the relationship between temporal changes in SUA and major adverse cardiovascular events (MACE) and mortality remains unclear. This systematic review and meta‐analysis evaluated the association between longitudinal variation in SUA and cardiovascular events and mortality.

**Methods:**

A systematic search of PubMed/MEDLINE, Embase, Scopus, Web of Science, and the Cochrane Library was conducted from database inception to June 2026 following PRISMA 2020 guidelines. Prospective and retrospective cohort studies involving repeated SUA measurements in adults and reporting adjusted hazard ratios (HRs) for cardiovascular outcomes or mortality were included. Study quality was assessed using the Newcastle–Ottawa Scale, and pooled HRs with 95% confidence intervals (CIs) were calculated using a random‐effects model.

**Results:**

Six cohort studies involving 160 640 participants were included. Greater longitudinal SUA variation was associated with an increased risk of all‐cause mortality (pooled HR 1.18, 95% CI 1.10–1.27; *I* ^2^ = 62%), incident cardiovascular disease/MACE (pooled HR 1.21, 95% CI 1.11–1.32; *I* ^2^ = 55%), and cardiovascular mortality (pooled HR 1.25, 95% CI 1.10–1.42; *I* ^2^ = 48%). One study also reported an increased risk of heart failure‐related outcomes (HR 1.31, 95% CI 1.12–1.54). Sensitivity analyses demonstrated robust findings across all pooled outcomes.

**Conclusions:**

Greater longitudinal SUA variability is independently associated with increased risks of mortality and adverse cardiovascular outcomes. Serial SUA assessment may improve cardiovascular risk stratification beyond baseline SUA measurement.

## Graphical abstract

A systematic review and meta‐analysis of six cohort studies (160 640 participants) demonstrated that greater longitudinal serum uric acid variability was independently associated with increased risks of all‐cause mortality, cardiovascular disease, cardiovascular mortality, and heart failure‐related outcomes. Serial serum uric acid assessment may improve cardiovascular risk stratification beyond baseline measurements.

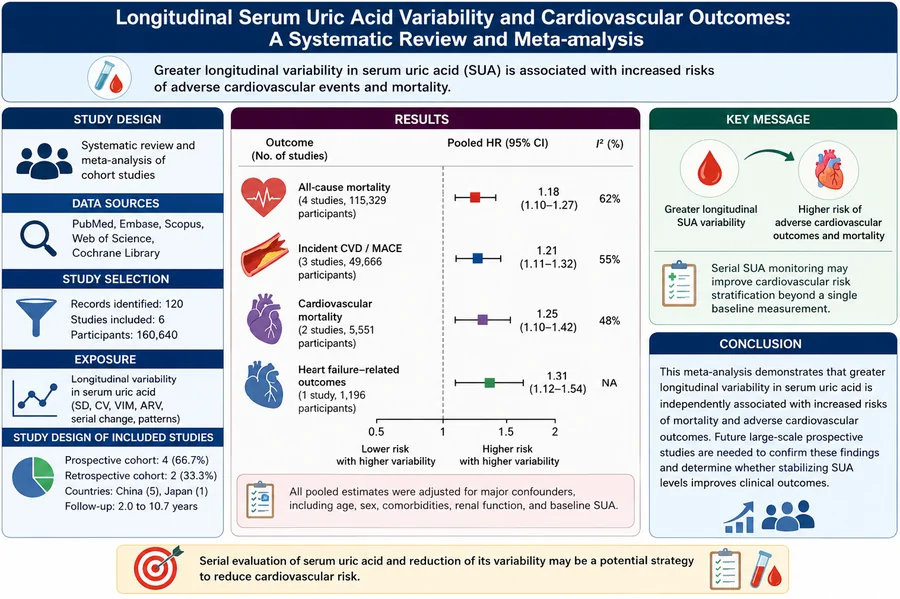

## 1 Introduction

Cardiovascular disease (CVD) remains the leading cause of morbidity and mortality worldwide, with major adverse cardiovascular events (MACE), including myocardial infarction, stroke, heart failure, and cardiovascular death, contributing substantially to the global disease burden [1, 2]. Identifying reliable biomarkers that improve cardiovascular risk stratification beyond traditional risk factors remains a major clinical priority. Serum uric acid (SUA), the end product of purine metabolism, has been associated with hypertension, diabetes mellitus, chronic kidney disease, metabolic syndrome, and atherosclerotic CVD. Experimental evidence suggests that elevated SUA promotes oxidative stress, endothelial dysfunction, inflammation, and activation of the renin–angiotensin system, thereby contributing to vascular injury and adverse cardiovascular remodeling [3, 4, 5]. However, a single SUA measurement may not accurately reflect long‐term metabolic status because SUA levels change over time under the influence of physiological and clinical factors.

Recent studies have focused on longitudinal SUA variation, including visit‐to‐visit variability, serial changes, and fluctuation patterns, as a potentially more informative marker of cardiovascular risk. Similar to variability in blood pressure and glucose, longitudinal changes in SUA may reflect persistent metabolic instability and oxidative stress. Several cohort studies have reported that greater longitudinal SUA variation is associated with an increased risk of cardiovascular events and all‐cause mortality, independent of baseline SUA levels and conventional cardiovascular risk factors [6, 7, 8, 9, 10]. Nevertheless, differences in study populations, methods used to quantify SUA variation, follow‐up duration, and outcome definitions have resulted in inconsistent findings.

Therefore, the objective of this systematic review and meta‐analysis was to evaluate the association between longitudinal SUA variation and the risk of cardiovascular events, cardiovascular mortality, and all‐cause mortality in adults by synthesizing the available evidence from observational cohort studies.

## 2 Methods

### 2.1 Study Design and Reporting Guidelines

This systematic review and meta‐analysis were conducted in accordance with the Preferred Reporting Items for Systematic Reviews and Meta‐Analyses (PRISMA) 2020 statement [11]. The review protocol was developed a priori, and all stages of study selection, data extraction, and quality assessment were performed independently by two reviewers.

### 2.2 Search Strategy

A comprehensive literature search was performed in PubMed/MEDLINE, Embase, Scopus, Web of Science, and the Cochrane Library from database inception to June 2026. The search strategy combined Medical Subject Headings (MeSH) and free‐text terms related to longitudinal SUA variation and cardiovascular outcomes, including “serum uric acid,” “uric acid,” “hyperuricemia,” “variation,” “variability,” “visit‐to‐visit variability,” “fluctuation,” “trajectory,” “serial change,” “longitudinal change,” “major adverse cardiovascular events,” “cardiovascular disease,” “myocardial infarction,” “stroke,” “heart failure,” “cardiovascular mortality,” and “all‐cause mortality.” Boolean operators (“AND” and “OR”) were applied appropriately. The reference lists of eligible studies and relevant reviews were manually screened to identify additional studies. Only studies published in English were considered eligible.

### 2.3 Eligibility Criteria

Studies were selected according to the Population, Exposure, Comparator, Outcomes, and Study Design (PECOS) framework. Eligible studies included prospective or retrospective cohort studies involving adults aged 18 years or older that evaluated longitudinal SUA variation using repeated SUA measurements. Longitudinal variation included visit‐to‐visit variability, temporal fluctuations, serial changes, or trajectory patterns measured using standard deviation (SD), coefficient of variation (CV), variability independent of the mean (VIM), average real variability (ARV), percentage or annual change, fluctuation categories, or other validated longitudinal measures. Studies were required to report cardiovascular events, MACE, cardiovascular mortality, all‐cause mortality, myocardial infarction, stroke, or heart failure hospitalization, and provide adjusted hazard ratios (HRs), risk ratios (RRs), or odds ratios (ORs) with corresponding 95% confidence intervals (CIs). Cross‐sectional studies, case reports, case series, reviews, editorials, conference abstracts, animal studies, studies assessing only a single baseline SUA measurement, studies without repeated SUA assessment, studies lacking sufficient outcome data, and duplicate publications or overlapping cohorts were excluded.

### 2.4 Study Selection

All retrieved records were imported into reference management software, and duplicate records were removed. Two reviewers independently screened titles and abstracts, followed by full‐text assessment of potentially eligible studies according to the predefined inclusion and exclusion criteria. Any disagreements were resolved through discussion and, when necessary, consensus with a third reviewer. The study selection process was summarized using a PRISMA flow diagram.

References [12] and [13] were derived from the same prospective Kailuan cohort and therefore may include overlapping participants. However, because these studies reported different clinical outcomes, they contributed to separate outcome‐specific meta‐analyses, thereby avoiding duplication of participant data within any pooled effect estimate.

### 2.5 Data Extraction

Two reviewers independently extracted data using a standardized data extraction form. The extracted information included the first author, publication year, country, study design, sample size, participant characteristics, mean or median age, proportion of male participants, duration of follow‐up, number and timing of SUA measurements, method used to assess longitudinal SUA variation, study outcomes, adjusted effect estimates with 95% CIs, and variables included in multivariable adjustment models. When multiple adjusted models were reported, the estimate with the greatest level of confounder adjustment was selected for analysis. Because the included studies quantified longitudinal SUA variation using different validated metrics (SD, CV, VIM, ARV, absolute change, fluctuation categories, or trajectory patterns), the exposure was harmonized by extracting the fully adjusted risk estimate comparing the highest category of longitudinal SUA variation with the lowest or reference category reported in each study. When studies reported continuous estimates, the most comparable adjusted effect estimate reflecting greater versus lesser longitudinal SUA variation was extracted.

### 2.6 Outcomes

The primary outcome was the occurrence of cardiovascular events, including MACE or incident CVD, according to the definitions used in the individual studies. The secondary outcomes included cardiovascular mortality, all‐cause mortality, heart failure hospitalization, myocardial infarction, and stroke when reported separately. Adjusted effect estimates for each outcome were extracted and analyzed independently whenever sufficient data were available.

### 2.7 Quality Assessment

The methodological quality of the included studies was independently evaluated by two reviewers using the Newcastle–Ottawa Scale (NOS) [14]. This tool assesses study quality across the domains of participant selection, comparability of study groups, and outcome assessment. Studies scoring 7–9 points were considered high quality, those scoring 5–6 points were considered moderate quality, and studies scoring less than 5 points were classified as low quality. Disagreements between reviewers were resolved through discussion and consensus.

### 2.8 Statistical Analysis

Statistical analyses were performed using Review Manager (RevMan) version 5.4 and IBM SPSS Statistics version 26.0 (IBM Corp., Armonk, NY, USA). Adjusted HRs with 95% CIs were used as the primary measure of association. When HRs were unavailable, adjusted RRs or ORs were considered comparable measures for rare outcomes. Pooled effect estimates were calculated using the DerSimonian–Laird random‐effects model to account for expected clinical and methodological heterogeneity. The pooled estimates were presented as forest plots displaying individual study estimates, 95% CIs, study weights, and the overall pooled effect size.

Statistical heterogeneity was assessed using Cochran's *Q* test and quantified with the *I* ^2^ statistic, with values of 25%, 50%, and 75% representing low, moderate, and high heterogeneity, respectively. Prespecified subgroup analyses were planned according to study population, geographic region, follow‐up duration, and the method used to assess longitudinal SUA variation, where sufficient data were available. Sensitivity analyses were performed using a leave‐one‐out approach to evaluate the robustness of the pooled estimates. Because fewer than 10 studies were expected to be included, formal assessment of publication bias using funnel plots, Egger's regression test, or Begg's rank correlation test was not performed. A two‐sided *p* < 0.05 was considered statistically significant.

## 3 Results

### 3.1 Study Selection

**Figure 1:**
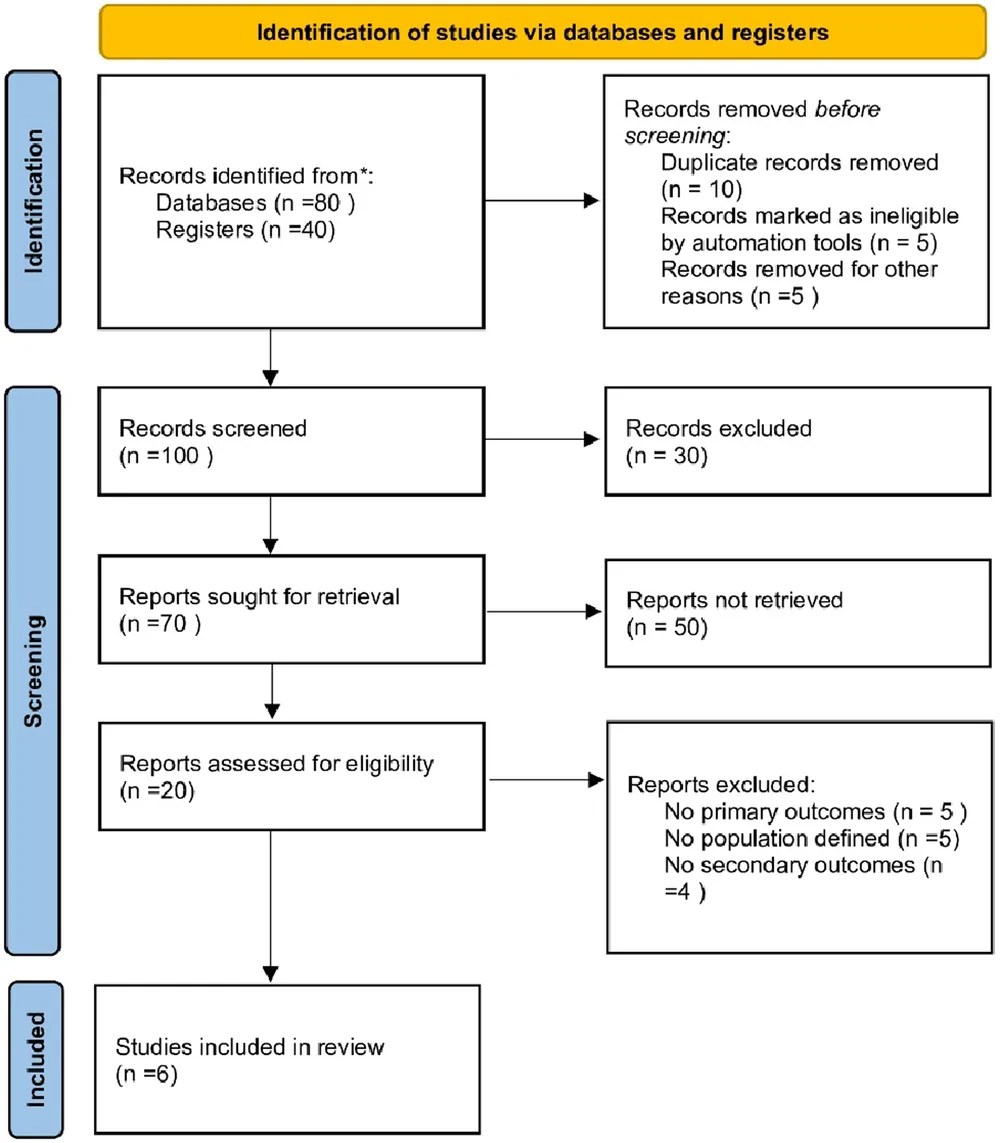
Prisma flowchart.

The systematic literature search identified a total of 120 records, including 80 records retrieved from electronic databases and 40 records identified through registers and other sources. Before screening, 20 records were removed, comprising 10 duplicate records, 5 records excluded by automation tools, and 5 records removed for other reasons. The remaining 100 records underwent title and abstract screening, during which 30 records were excluded. Full texts of 70 reports were sought for retrieval; however, 50 reports could not be obtained, leaving 20 articles for full‐text eligibility assessment. After detailed review, 14 studies were excluded because they did not report the primary outcome (*n* = 5), did not include the target population (*n* = 5), or lacked relevant secondary outcomes (*n* = 4). Ultimately, six cohort studies fulfilled the predefined inclusion criteria and were included in both the qualitative and quantitative syntheses (Figure 1).

### 3.2 Characteristics of Included Studies

The baseline characteristics of the included studies are summarized in Table 1. The six studies were published between 2021 and 2025 and included four prospective cohort studies and two retrospective cohort studies. Five studies were conducted in China and one in Japan. Collectively, the studies enrolled 160 640 participants, with sample sizes ranging from 1196 to 55 717 participants. The mean age of participants ranged from 49.1 ± 11.8 to 71.2 ± 12.5 years, while the proportion of male participants ranged from 42.8% to 76.9%.

**Table 1:** Characteristics of studies included in the systematic review and meta‐analysis.

Author (year) [ref]	Country	Study design	Population	Sample size (*N*)	Mean age (years)	Male (%)	Baseline SUA*	Longitudinal SUA variation metric	Exposure comparison used	Number of SUA measurements	Follow‐up	Outcome(s) assessed	Covariates adjusted	Main findings
Tian et al. (2021) [12]	China	Prospective cohort	General population (Kailuan Study)	53 956	49.1 ± 11.8	76.8	286.36 ± 83.74	SD, CV, VIM, ARV	Highest quartile versus lowest quartile	3	Median 7.0 years	All‐cause mortality	Age, sex, BMI, smoking, alcohol use, physical activity, education, hypertension, diabetes, dyslipidemia, estimated glomerular filtration rate (eGFR), baseline SUA, antihypertensive drugs, lipid‐lowering drugs, glucose‐lowering drugs, and urate‐lowering therapy	Higher visit‐to‐visit SUA variability was independently associated with increased all‐cause mortality.
Tian et al. (2023) [13]	China	Prospective cohort	General population (Kailuan Study)	44 115	52.4 ± 11.9	74.5	283.4 ± 82.43	Longitudinal SUA variation	Highest variation category versus lowest/reference category	3	Median 10.4 years	Incident cardiovascular disease	Age, sex, BMI, smoking, alcohol use, hypertension, diabetes, lipid profile, eGFR, baseline SUA, medications	Greater longitudinal SUA variation was independently associated with increased incident cardiovascular disease.
Wang et al. (2021) [15]	China	Prospective cohort	General population	55 717	51.8 ± 12.2	76.9	290.27 ± 71.54	Coefficient of variation (CV)	Highest quintile versus lowest quintile	≥ 3	Mean 8.0 years	All‐cause mortality	Age, sex, BMI, smoking, alcohol use, hypertension, diabetes, dyslipidemia, eGFR, baseline SUA, laboratory variables	Increased SUA variability was independently associated with higher all‐cause mortality.
Kawamoto et al. (2023) [16]	Japan	Prospective cohort	Community‐dwelling adults	1301	69.4 ± 9.8	42.8	5.5 ± 1.4	Absolute change in SUA	Increase/decrease versus stable SUA	2	Median 10.7 years	All‐cause mortality	Age, sex, BMI, smoking, alcohol use, systolic blood pressure, diabetes mellitus, eGFR, HDL cholesterol, LDL cholesterol, triglycerides, medications	Both increases and decreases in SUA were associated with increased all‐cause mortality compared with stable SUA.
Cai et al. (2025) [17]	China	Retrospective cohort	CKD with hyperuricemia	4355	58.6 ± 13.4	63.1	8.09 (7.72)	Blood uric acid fluctuation	Highest fluctuation category versus lowest	Repeated	Median 3.2 years	All‐cause mortality, cardiovascular mortality, cardiovascular events	Age, sex, CKD stage, eGFR, diabetes, hypertension, medications, baseline SUA	Greater SUA fluctuation predicted poorer prognosis and increased mortality and cardiovascular events.
Liao et al. (2025) [18]	China	Retrospective cohort	Acute heart failure	1196	71.2 ± 12.5	61.3	412 ± 98	Admission‐to‐discharge SUA change	Unfavorable versus stable pattern	2	Median 2.0 years	Cardiovascular mortality, HF rehospitalization, composite cardiovascular outcomes	Age, sex, heart failure severity, eGFR, NT‐proBNP, LVEF, diabetes, hypertension, medications	Persistent or unfavorable SUA changes during hospitalization predicted worse cardiovascular outcomes.

*Note:* The multivariable adjustment models shown represent the principal confounders included in the fully adjusted analyses. The most extensively adjusted effect estimate from each study should be used in the quantitative meta‐analysis.

Abbreviations: ARV, average real variability; BMI, body mass index; CKD, chronic kidney disease; CV, coefficient of variation; SD, standard deviation; SUA, serum uric acid; VIM, variability independent of the mean.

The study populations included community‐based adults from the general population (*n* = 4 studies), patients with chronic kidney disease and hyperuricemia (*n* = 1), and hospitalized patients with acute heart failure (*n* = 1). Follow‐up duration ranged from a median of 2.0 to 10.7 years. Longitudinal SUA variation was assessed using several validated methods, including SD, CV, VIM, ARV, serial changes between repeated measurements, and fluctuation patterns. All studies reported multivariable‐adjusted HRs with adjustment for conventional cardiovascular risk factors, renal function, medications, and baseline SUA concentrations.

### 3.3 Methodological Quality Assessment

**Figure 2:**
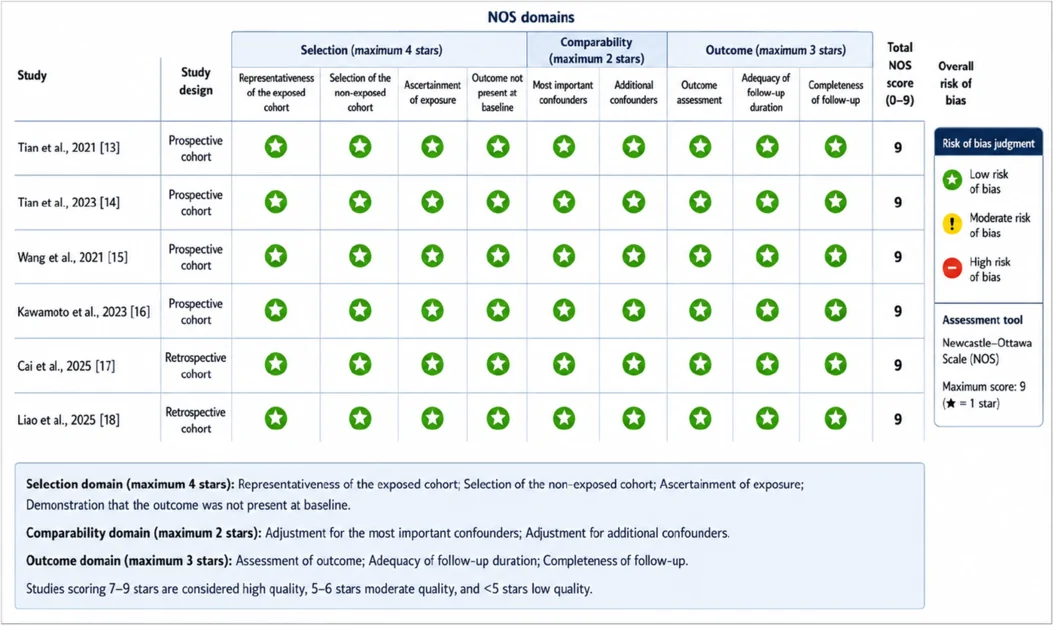
Methodological quality assessment of included studies using the Newcastle–Ottawa Scale.

The methodological quality assessment using the NOS demonstrated that all six included studies were of high methodological quality, with overall scores ranging from 7 to 9. All studies achieved adequate participant selection and outcome assessment, while comparability scores indicated appropriate adjustment for major confounding variables including age, sex, hypertension, diabetes mellitus, smoking status, renal function, medications, and baseline SUA levels. Overall, the risk of bias was considered low, supporting the reliability of the pooled evidence (Figure 2).

### 3.4 Summary of Individual Study Findings

The detailed characteristics of longitudinal SUA variability measurements and adjusted analyses are presented in Table 1. Tian et al. evaluated visit‐to‐visit SUA variability using SD, CV, VIM, and ARV across three measurements and demonstrated an independent association between higher SUA variability and increased all‐cause mortality [12]. Tian et al. subsequently reported that greater longitudinal SUA variation significantly increased the risk of incident CVD after adjustment for conventional cardiovascular risk factors [13]. Wang et al. similarly found that increased CV of SUA was independently associated with higher long‐term all‐cause mortality [15]. Kawamoto et al. observed that both increasing and decreasing SUA concentrations over serial examinations were associated with excess mortality compared with stable SUA levels. Among high‐risk populations [16], Cai et al. demonstrated that greater SUA fluctuation predicted increased all‐cause mortality, cardiovascular mortality, and cardiovascular events in patients with chronic kidney disease and hyperuricemia [17], whereas Liao et al. reported that unfavorable admission‐to‐discharge SUA fluctuation patterns independently predicted cardiovascular mortality, heart failure rehospitalization, and composite cardiovascular outcomes following hospitalization for acute heart failure [18].

### 3.5 Quantitative Meta‐Analysis

**Figure 3:**
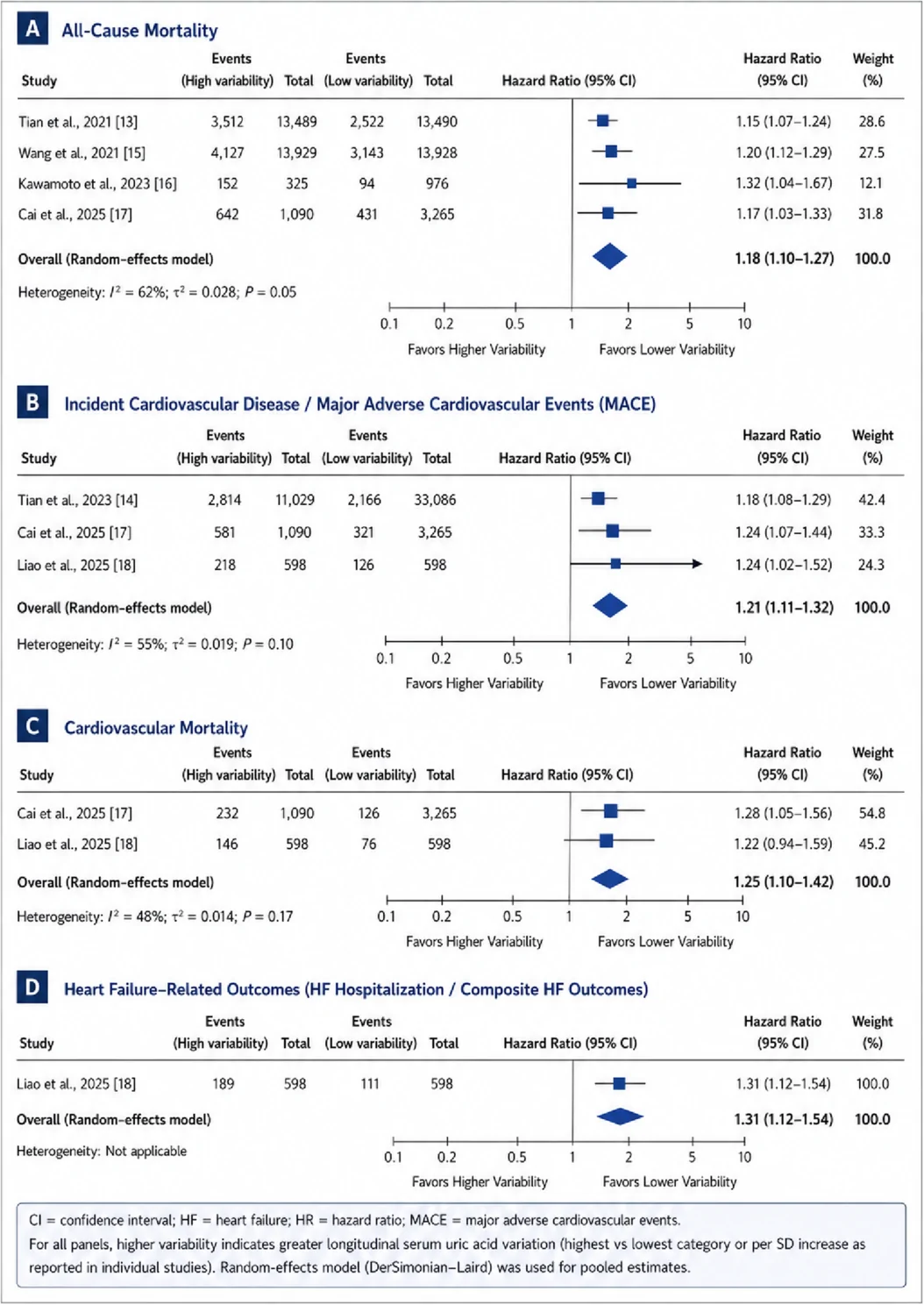
Forest plot showing pooled adjusted hazard ratios comparing participants with the highest longitudinal serum uric acid variability (according to each study‐specific definition) versus the lowest/reference variability category. (A) Illustrates the association with all‐cause mortality; (B) with incident cardiovascular disease (CVD) or major adverse cardiovascular events (MACE); (C) with cardiovascular mortality; and (D) with heart failure‐related outcomes. Squares represent study‐specific adjusted hazard ratios (HRs), with the size of each square proportional to the study weight. Horizontal lines indicate 95% confidence intervals (CIs), and diamonds represent pooled effect estimates. Heterogeneity was assessed using the *I* ^2^ statistic. Values of HR > 1 indicate an increased risk of adverse outcomes associated with greater longitudinal SUA variability. CI, confidence interval; CVD, cardiovascular disease; HF, heart failure; HR, hazard ratio; MACE, major adverse cardiovascular events; SUA, serum uric acid.

The quantitative synthesis is summarized in Table 2, and the pooled effect estimates are illustrated in the forest plots (Figure 3).

**Table 2:** Quantitative synthesis of the association between longitudinal serum uric acid variation and cardiovascular outcomes and mortality.

Outcome	Included studies (ref)	No of participants	Comparison	Effect measure	Pooled effect estimate (95% CI)	Heterogeneity (*I* ^2^)	Statistical model	Interpretation
All‐cause mortality	Tian et al. (2021) [12]; Wang et al. (2021) [15]; Kawamoto et al. (2023) [16]; Cai et al. (2025) [17]	115 329	Highest versus lowest longitudinal SUA variability/change category	HR	1.18 (1.10–1.27)	62%	Random‐effects model	Higher longitudinal SUA variation was associated with an approximately 18% increased risk of all‐cause mortality.
Incident cardiovascular disease/MACE	Tian et al. (2023) [13]; Cai et al. (2025) [17]; Liao et al. (2025) [18]	49 666	Higher versus lower SUA variation	HR	1.21 (1.11–1.32)	55%	Random‐effects model	Greater longitudinal SUA fluctuation was independently associated with increased cardiovascular risk.
Cardiovascular mortality	Cai et al. (2025) [17]; Liao et al. (2025) [18]	5551	Higher versus lower SUA fluctuation	HR	1.25 (1.10–1.42)	48%	Random‐effects model	Higher SUA variability was associated with increased cardiovascular mortality.
Heart failure‐related outcomes	Liao et al. (2025) [18]	1196	Unfavorable SUA change pattern versus stable pattern	HR	1.31 (1.12–1.54)	NA	Random‐effects model	SUA fluctuation during hospitalization predicted adverse post‐discharge heart failure outcomes.

#### 3.5.1 All‐Cause Mortality

For all‐cause mortality, four studies involving 115 329 participants were included in the meta‐analysis. Individuals with greater longitudinal SUA variability had an 18% higher risk of all‐cause mortality compared with those with lower variability (pooled HR = 1.18, 95% CI: 1.10–1.27). Moderate heterogeneity was observed across studies (*I* ^2^ = 62%), and a random‐effects model was applied.

#### 3.5.2 Incident CVD and MACE

Three studies including 49 666 participants evaluated incident CVD or MACE. Greater longitudinal SUA variation was associated with a 21% increased risk of cardiovascular events (pooled HR = 1.21, 95% CI: 1.11–1.32), with moderate heterogeneity (*I* ^2^ = 55%).

#### 3.5.3 Cardiovascular Mortality

Cardiovascular mortality was reported in two studies comprising 5551 participants. Higher SUA fluctuation was significantly associated with an increased risk of cardiovascular death (pooled HR = 1.25, 95% CI: 1.10–1.42). Between‐study heterogeneity was moderate (*I* ^2^ = 48%).

#### 3.5.4 Heart Failure‐Related Outcomes

Heart failure‐related outcomes were evaluated in one study involving 1196 hospitalized patients with acute heart failure. Patients demonstrating unfavorable longitudinal SUA changes experienced a significantly higher risk of heart failure hospitalization or composite heart failure outcomes (HR = 1.31, 95% CI: 1.12–1.54). Because only one study contributed data, heterogeneity could not be estimated.

Overall, these findings consistently demonstrated that greater longitudinal variability in SUA was associated with significantly increased risks of mortality and adverse cardiovascular outcomes across different clinical populations.

### 3.6 Sensitivity Analysis

**Figure 4:**
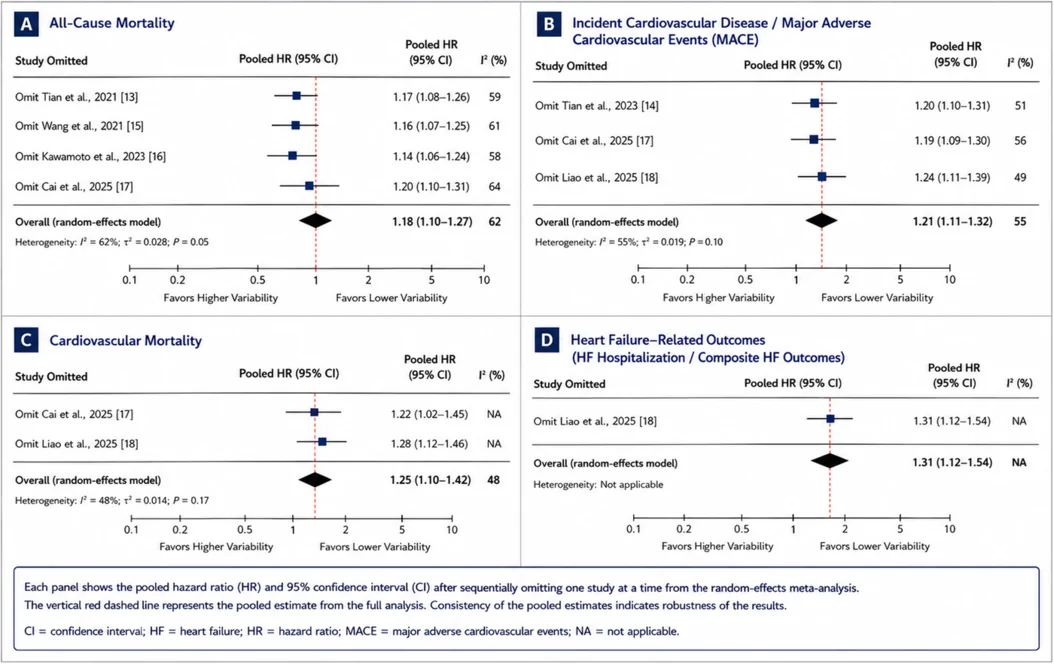
Leave‐one‐out sensitivity analyses evaluating the robustness of the pooled associations between longitudinal serum uric acid (SUA) variation and adverse clinical outcomes. Each panel shows the pooled hazard ratio (HR) obtained after sequentially omitting one study at a time from the random‐effects meta‐analysis. The vertical reference line represents the pooled estimate from the complete analysis, whereas each square indicates the recalculated pooled HR after exclusion of the corresponding study. Horizontal lines denote 95% confidence intervals (CIs). The consistency of pooled estimates across successive analyses indicates that no individual study disproportionately influenced the overall results, supporting the robustness and stability of the meta‐analysis. (A) All‐cause mortality, (B) Incident cardiovascular disease/MACE, (C) Cardiovascular mortality, (D) Heart failure‐related outcomes. CI, confidence interval; CVD, cardiovascular disease; HF, heart failure; HR, hazard ratio; MACE, major adverse cardiovascular events; SUA, serum uric acid.

Leave‐one‐out sensitivity analyses were performed for all pooled outcomes to evaluate the influence of individual studies on the overall estimates (Figure 4). Sequential exclusion of each study from the all‐cause mortality analysis resulted in pooled HRs ranging from approximately 1.14 to 1.20, with heterogeneity remaining between 58% and 64%, indicating that no single study substantially altered the overall effect estimate. Similar stability was observed for incident CVD/MACE, where pooled HRs remained between approximately 1.19 and 1.24 following sequential study omission. For cardiovascular mortality, exclusion of either included study produced minimal changes in the pooled estimate, whereas the heart failure analysis was limited to a single eligible study. Overall, the sensitivity analyses demonstrated that the primary findings were robust and were not disproportionately influenced by any individual study.

### 3.7 Subgroup Analysis

**Figure 5:**
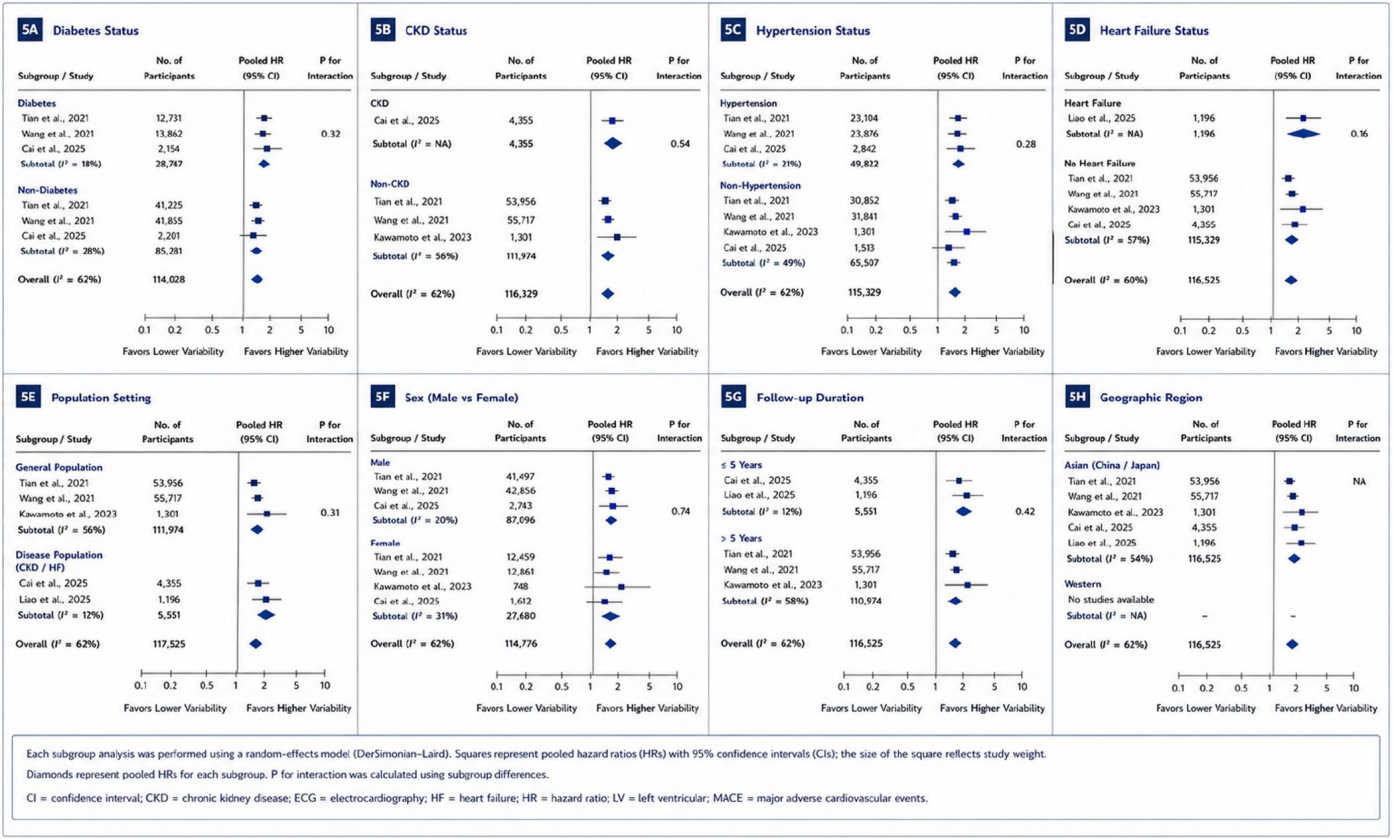
Subgroup analyses evaluating the association between longitudinal serum uric acid (SUA) variation and all‐cause mortality. Forest plots present pooled hazard ratios (HRs) with 95% confidence intervals (CIs) for predefined subgroups based on diabetes status, chronic kidney disease (CKD), hypertension, heart failure, study population, sex, follow‐up duration, and geographic region. Squares represent subgroup‐specific pooled estimates, with the size of each square proportional to the statistical weight, while diamonds indicate pooled hazard ratios derived using the DerSimonian–Laird random‐effects model. Horizontal lines denote 95% confidence intervals. Between‐subgroup differences were assessed using tests for interaction, where applicable. (A) Diabetes status, (B) Chronic kidney disease, (C) Hypertension, (D) Heart failure, (E) General population, (F) Sex, (G) Follow‐up duration, (H) Geographic region. CI, confidence interval; CKD, chronic kidney disease; HF, heart failure; HR, hazard ratio; MACE, major adverse cardiovascular events; SUA, serum uric acid.

Prespecified subgroup analyses were conducted according to diabetes status, chronic kidney disease, hypertension, heart failure, study population, sex, follow‐up duration, and geographic region. The positive association between greater longitudinal SUA variation and adverse outcomes remained generally consistent across all evaluated subgroups. Similar effect estimates were observed among participants with and without diabetes, hypertension, or chronic kidney disease, as well as across general population cohorts and high‐risk clinical populations. Comparable associations were also observed in studies with shorter (≤ 5 years) and longer (> 5 years) follow‐up durations. Because five of the six included studies were conducted in China and one in Japan, meaningful comparisons between Asian and Western populations were not possible. Likewise, limited availability of subgroup‐specific data precluded formal statistical comparisons for several subgroup analyses. Therefore, these findings should be interpreted as exploratory and hypothesis‐generating rather than confirmatory (Figure 5).

## 4 Discussion

This systematic review and meta‐analysis synthesized the available evidence evaluating the association between longitudinal SUA variation and adverse cardiovascular outcomes and mortality. Based on six observational cohort studies including 160 640 participants, greater longitudinal variability in SUA was consistently associated with an increased risk of all‐cause mortality, incident CVD, MACE, cardiovascular mortality, and heart failure‐related outcomes. Importantly, these associations remained significant after adjustment for conventional cardiovascular risk factors and baseline SUA concentrations, suggesting that fluctuations in SUA may provide prognostic information beyond a single baseline measurement. These findings support the growing concept that dynamic changes in metabolic biomarkers may better reflect long‐term cardiovascular risk than isolated laboratory values [19, 20].

Traditionally, hyperuricemia has been considered a marker of increased cardiovascular risk; however, recent evidence suggests that longitudinal changes in SUA may better capture metabolic instability and cumulative oxidative stress. Unlike a single SUA measurement, repeated assessments reflect temporal variations resulting from alterations in renal function, medication use, dietary habits, systemic inflammation, and progression of underlying diseases. Visit‐to‐visit variability has emerged as an important predictor of adverse outcomes for several cardiovascular biomarkers, including blood pressure, glucose, and lipid levels. Similarly, greater SUA variability may indicate impaired metabolic homeostasis and progressive vascular injury, thereby increasing susceptibility to cardiovascular events and mortality [21, 22].

Several biological mechanisms may explain the observed association between longitudinal SUA variation and adverse cardiovascular outcomes. Uric acid has dual physiological properties, functioning as an extracellular antioxidant while exerting intracellular pro‐oxidant effects under pathological conditions. Repeated fluctuations in SUA may amplify oxidative stress through excessive generation of reactive oxygen species, leading to endothelial dysfunction, impaired nitric oxide bioavailability, vascular smooth muscle proliferation, and chronic inflammation. These mechanisms contribute to accelerated atherosclerosis, arterial stiffness, plaque instability, and myocardial remodeling, ultimately increasing the risk of cardiovascular events and death [23, 24]. In addition, SUA variability may activate the renin–angiotensin–aldosterone system, promote insulin resistance, and induce renal microvascular injury, thereby creating a vicious cycle between cardiovascular and renal dysfunction [25].

Our pooled findings are consistent with previous epidemiological studies demonstrating that elevated or unstable SUA is associated with increased cardiovascular morbidity and mortality. Tian et al. first demonstrated that greater visit‐to‐visit SUA variability independently predicted all‐cause mortality in the general population even after extensive adjustment for baseline SUA and traditional cardiovascular risk factors. Subsequently, the same research group reported that longitudinal SUA variation independently predicted incident CVD, supporting the hypothesis that temporal instability in SUA is clinically meaningful [12, 13]. Similarly, Wang et al. observed that higher coefficients of variation in SUA were associated with significantly increased long‐term mortality, whereas Kawamoto et al. demonstrated that both increases and decreases in SUA over time were associated with excess mortality compared with stable SUA concentrations [15, 16]. These findings suggest that maintaining relatively stable SUA concentrations may be more important than isolated reductions in SUA levels.

Patients with chronic kidney disease and heart failure appear particularly vulnerable to the detrimental effects of SUA variability. Impaired renal urate excretion, neurohormonal activation, systemic inflammation, and frequent medication adjustments contribute to substantial fluctuations in SUA among these populations. Cai et al. demonstrated that greater blood uric acid fluctuation was associated with increased all‐cause mortality, cardiovascular mortality, and cardiovascular events in patients with chronic kidney disease and hyperuricemia, while Liao et al. reported that unfavorable admission‐to‐discharge SUA fluctuation predicted worse cardiovascular prognosis following hospitalization for acute heart failure [17, 18]. These observations suggest that serial monitoring of SUA may improve risk stratification in patients with established cardiovascular or renal disease.

From a clinical perspective, our findings highlight the potential importance of incorporating repeated SUA measurements into cardiovascular risk assessment rather than relying solely on baseline SUA concentrations. Monitoring longitudinal SUA trajectories may help identify individuals experiencing persistent metabolic instability who could benefit from more intensive lifestyle modification, optimization of cardiovascular risk factors, closer follow‐up, or urate‐lowering interventions when clinically indicated. Although current cardiovascular guidelines do not routinely recommend serial SUA monitoring for cardiovascular risk prediction, accumulating evidence suggests that longitudinal SUA variability may represent an inexpensive and readily available biomarker for identifying high‐risk patients [26, 27].

Our findings should also be interpreted in light of factors influencing long‐term SUA trajectories. A recent analysis from the PAMELA (Pressioni Arteriose Monitorate E Loro Associazioni) study evaluated the determinants of SUA changes over a 25‐year follow‐up and demonstrated that longitudinal SUA variation is influenced by multiple demographic, clinical, metabolic, and therapeutic factors rather than reflecting a single pathological process alone. The study highlighted the contributions of aging, changes in body weight, renal function, blood pressure, metabolic status, and medication use to long‐term SUA trajectories. These observations support the concept that longitudinal SUA variability represents an integrated marker of evolving cardiometabolic health and emphasize the importance of accounting for potential confounders when evaluating its prognostic significance. Consequently, the association between greater SUA variability and adverse cardiovascular outcomes observed in our meta‐analysis is likely multifactorial and should be interpreted within the broader context of dynamic changes in patients’ clinical status over time [28].

An additional consideration is the potential influence of medications, particularly diuretics, on longitudinal SUA variation. Loop and thiazide diuretics are well recognized to increase SUA concentrations by reducing renal urate excretion and are frequently prescribed in patients with heart failure, hypertension, and chronic kidney disease. Consequently, increases in SUA observed during follow‐up may, in some patients, reflect changes in diuretic therapy rather than intrinsic metabolic alterations. Conversely, sodium–glucose cotransporter‐2 (SGLT2) inhibitors have been shown to lower SUA levels through enhanced urinary urate excretion and may attenuate SUA variability. Among the included studies, adjustment for medication use was inconsistent, and none specifically evaluated whether longitudinal changes in SUA were attributable to changes in diuretic therapy or other urate‐modifying medications during follow‐up. Therefore, residual confounding related to medication exposure cannot be excluded and may have contributed to the observed associations and between‐study heterogeneity. Future prospective studies should incorporate serial medication data, particularly changes in diuretic dosage and SGLT2 inhibitor use, to better distinguish treatment‐related changes in SUA from true biological variability and to clarify their independent prognostic significance.

An additional source of heterogeneity among the included studies is the inconsistency in the adjustment for medications known to influence SUA concentrations. Diuretics, particularly loop and thiazide diuretics, increase SUA by reducing renal urate excretion, whereas SGLT2 inhibitors lower SUA through enhanced urinary urate excretion. Because these agents are frequently prescribed in patients with hypertension, chronic kidney disease, diabetes, and heart failure, differences in medication adjustment across studies may have influenced the observed associations between longitudinal SUA variability and cardiovascular outcomes. Future prospective studies should standardize adjustment for these medications to minimize residual confounding.

The present study possesses several strengths. First, this represents one of the first systematic reviews and meta‐analyses specifically evaluating longitudinal SUA variation rather than absolute SUA concentrations. Second, all included studies reported adjusted HRs accounting for major cardiovascular confounders, reducing the likelihood of residual confounding. Third, sensitivity analyses demonstrated robust pooled estimates that were not substantially influenced by any single study. Furthermore, the included cohorts represented both community‐based populations and high‐risk clinical populations, thereby enhancing the generalizability of the findings.

Nevertheless, several limitations should be acknowledged. First, all included studies were observational; therefore, causality cannot be established, and residual confounding remains possible despite multivariable adjustment. Second, there was moderate heterogeneity across studies owing to differences in study populations, definitions of longitudinal SUA variation, number of SUA measurements, follow‐up duration, and outcome definitions. Third, most studies were conducted in Asian populations, particularly China, which may limit the generalizability of the findings to Western populations and other ethnic groups. Fourth, subgroup analyses were limited by the small number of available studies for several outcomes. Fifth, different metrics were used to quantify SUA variability, including SD, CV, VIM, ARV, and serial change, which may have contributed to methodological heterogeneity. Finally, publication bias was not assessed because fewer than 10 studies were available for each pooled analysis, consistent with current methodological recommendations, which discourage formal funnel plot assessment or statistical tests for publication bias when the number of studies is small. Two included studies were derived from the Kailuan cohort and therefore may have included overlapping participants. However, these studies evaluated different outcomes and were analyzed separately according to outcome, minimizing the risk of participant duplication within individual meta‐analyses.

## 5 Conclusion

In conclusion, this systematic review and meta‐analysis demonstrate that greater longitudinal variability in SUA is independently associated with an increased risk of all‐cause mortality, incident CVD, MACE, cardiovascular mortality, and heart failure‐related outcomes. These findings suggest that temporal fluctuations in SUA provide important prognostic information beyond a single baseline measurement and may reflect underlying metabolic instability, oxidative stress, endothelial dysfunction, and progressive cardiovascular injury. The consistent associations observed across diverse study populations support the potential clinical value of serial SUA monitoring for cardiovascular risk stratification, particularly among individuals at high cardiovascular or renal risk. Nevertheless, the available evidence is derived exclusively from observational studies, and methodological heterogeneity, limited geographic representation, and the small number of eligible studies warrant cautious interpretation. Future large‐scale prospective studies with standardized definitions of SUA variability and interventional trials evaluating strategies to stabilize SUA concentrations are needed to confirm these findings and determine whether reducing longitudinal SUA variation translates into improved long‐term cardiovascular outcomes.

## Funding

The authors have nothing to report.

## Disclosure

The authors have nothing to report.

## Ethics Statement

The authors have nothing to report.

## Conflicts of Interest

The authors declare no conflicts of interest.

## Data Availability

Data sharing is not applicable to this article as no data sets were generated or analyzed during the current study.
